# Link between light-triggered Mg-banding and chamber formation in the planktic foraminifera *Neogloboquadrina dutertrei*

**DOI:** 10.1038/ncomms15441

**Published:** 2017-05-15

**Authors:** Jennifer S. Fehrenbacher, Ann D. Russell, Catherine V. Davis, Alexander C. Gagnon, Howard J. Spero, John B. Cliff, Zihua Zhu, Pamela Martin

**Affiliations:** 1 College of Earth, Ocean and Atmospheric Sciences, Oregon State University, Corvallis, Oregon 97331, USA; 2 Department of Earth and Planetary Sciences, University of California, Davis, California 95616, USA; 3 School of Oceanography, University of Washington, Seattle, Washington 98105, USA; 4 Environmental Molecular Sciences Laboratory, Pacific Northwest National Laboratory, Richland, Washington 99354, USA; 5 3726 Totem Ln, Indianapolis, Indiana 46208, USA

## Abstract

The relationship between seawater temperature and the average Mg/Ca ratios in planktic foraminifera is well established, providing an essential tool for reconstructing past ocean temperatures. However, many species display alternating high and low Mg-bands within their shell walls that cannot be explained by temperature alone. Recent experiments demonstrate that intrashell Mg variability in *Orbulina universa*, which forms a spherical terminal shell, is paced by the diurnal light/dark cycle. Whether Mg-heterogeneity is also diurnally paced in species with more complex shell morphologies is unknown. Here we show that high Mg/Ca-calcite forms at night in cultured specimens of the multi-chambered species *Neogloboquadrina dutertrei*. Our results demonstrate that *N. dutertrei* adds a significant amount of calcite, and nearly all Mg-bands, after the final chamber forms. These results have implications for interpreting patterns of calcification in *N. dutertrei* and suggest that diurnal Mg-banding is an intrinsic component of biomineralization in planktic foraminifera.

## Abstract (web summary)

The degree to which the diurnal light/dark cycle influences Mg-heterogeneity in foraminifera with complex shell morphologies remains unknown. Here, using highly spatially resolved analytical techniques, the authors investigate Mg-banding and calcification in isotope-labelled *Neogloboquadrina dutertrei* specimens.

The Mg/Ca ratios recorded in the calcite shells or ‘tests' of planktic foraminifera are widely used in paleoceanography to reconstruct past sea surface temperatures. The Mg/Ca-temperature proxy is based on empirical calibration studies, typically using bulk solution analytical methods, which demonstrate that temperature is the primary control over planktic foraminiferal Mg/Ca^1^,^2^. Seawater pH and salinity are secondary influences on shell Mg/Ca ratios in planktic foraminifera, but play a minor role in comparison to temperature^3^,^4^,^5^,^6^. On long time scales (>100 Ky) secular changes in seawater Mg/Ca composition can also have an impact on Mg/Ca-based paleotemperature reconstructions^7^,^8^. The utility of foraminiferal Mg/Ca in paleothermometry expanded when researchers combined Mg/Ca ratios with δ^18^O analyses to reconstruct both temperature and salinity and to explore changes in the hydrologic cycle^9^,^10^,^11^,^12^,^13^,^14^. Although foraminiferal Mg/Ca has since become one of the most common paleothermometry proxies in paleoceanographic reconstructions, research using micro- and nanoscale analytical methodologies demonstrate that Mg is heterogeneously distributed within the shell wall of many foraminifers, often occurring in high- and low-Mg-bands (Table 1). Although this heterogeneity is likely to be accounted for in empirical calibrations^15^, understanding what causes Mg heterogeneity is essential for proper interpretation of past temperature records and the fundamentals of foraminifera biomineralization.

Experimental data show that specimens of the symbiont-bearing species *Orbulina universa* grown under constant conditions (controlled temperature and seawater chemistry) precipitate thin layers of high Mg/Ca calcite at night and thicker layers of low Mg/Ca calcite during the day. Temperature and pH modulate the amplitude and absolute ratios of both the high- and low-Mg-bands^15^. However, *O. universa* is the only foraminifer species that forms a terminal shell of simple spherical geometry. Most foraminifera used in paleoceanographic reconstructions have more complex multi-chambered morphologies. It is unknown whether Mg/Ca heterogeneity in these multi-chambered species is also diurnally paced or the result of other growth processes.

In multi-chambered foraminifera, Mg-banding may be linked to chamber formation processes. In the classic model of chamber formation, a new chamber begins with the delineation of the chamber shape by a primary organic sheet (POS) and the formation of an organic template^16^,^17^,^18^,^19^,^20^. Calcite precipitation initially commences on both sides of the POS^21^,^22^. The chamber growth model proposes that a thin layer of calcite is added to the entire shell (that is, over all existing chambers) when a new chamber forms, and that layered calcification may represent 90% of the total calcite added during ontogeny^16^,^17^,^18^. This model is invoked to explain lamellar calcite layering, chamber-to-chamber differences in shell thickness and possibly the presence of Mg-banding^23^. If chamber formation processes were responsible for Mg-banding, we would expect to see more Mg-bands in chambers added during early ontogenetic growth compared to chambers formed during late ontogeny.

We hypothesize that Mg-banding is a fundamental component of foraminiferal calcification, and that Mg-banding in multi-chambered species is due to diurnal calcification cycles rather than to chamber formation processes. We investigate this hypothesis using cultured specimens of the non-spinose planktic foraminifer *Neogloboquadrina dutertrei*. *N. dutertrei* is a multi-chambered, thermocline-dwelling planktic foraminifer that possesses intracellular chrysophyte algal symbionts^24^. Fossil specimens of this species are commonly used to reconstruct thermocline hydrography^25^,^26^,^27^,^28^,^29^ and are known to display Mg-banding^30^. Observations that the chamber wall in fossil specimens of *N. dutertrei* also displays Mg-banding similar to spinose species such as *O. universa* suggests that a common mechanism may be responsible for Mg-banding in both species.

In this study, we use isotope-labelling culture techniques to evaluate controls on elemental heterogeneity. We grew specimens under controlled conditions (constant temperature, pH and salinity) and transferred the specimens into isotopically modified seawater on a 12 h:12 h day/night (light/dark) cycle. We also cultured ‘control' specimens that were transferred into isotopically modified seawater after collection, but did not undergo day/night transfers. We evaluate the timing of trace element variability in these cultured specimens using the highly spatially resolved analytical techniques Laser ablation inductively coupled plasma mass spectrometry (LA-ICP-MS) and NanoSIMS image mapping. We show that *N. dutertrei* adds nearly all high Mg-bands after the final chamber forms and that Mg-banding is paced by the diurnal light-cycle. Finally, we use these results to formulate a new chamber growth and crust addition model for *N. dutertrei* and extend a modified version of this growth model to the planktic foraminifer *O. universa*.

## Results

### NanoSIMS results for transfer specimens

**Figure 1:**
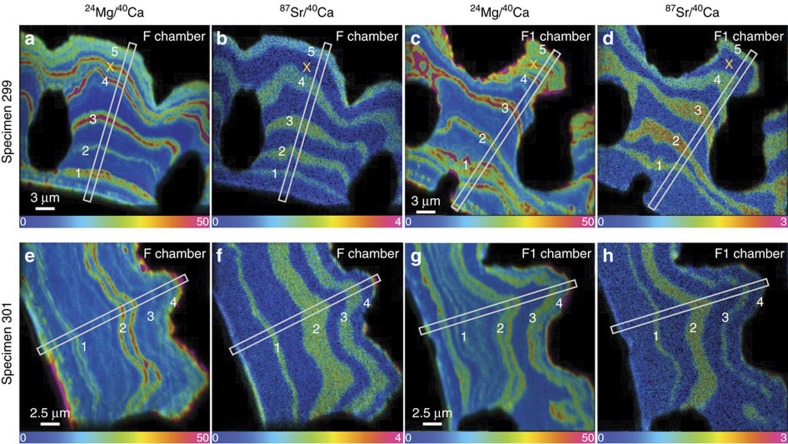
NanoSIMS images of raw count ratios ^24^Mg/^40^Ca and ^87^Sr/^40^Ca in the F and F1 chambers of two cultured *N. dutertrei* specimens. (**a**–**d**) Specimen 299. (**e**–**h**) Specimen 301. White boxes mark the locations where NanoSIMS line scan profiles were generated (Figs 2 and 3). The white numbers superimposed on the images mark the number of night transfers (for example, ‘1' is the first night transfer). The ‘*X*' in **a** through **d** denotes an Mg-band that was not labelled with ^87^Sr during the night hours, because the specimen was not transferred into ^87^Sr-labelled seawater that night. These images represent the summation of a stack of 30 NanoSIMS frames that were shift corrected; the hue scale bar represents the ratio of the summed ^24^Mg or ^87^Sr counts divided by ^40^Ca counts × 10,000.

LA-ICP-MS is used to identify suitable specimens for NanoSIMS analysis (Supplementary Discussion and Supplementary Fig. 1). Two specimens (299 and 301) added a significant amount of calcite in culture and were subsequently chosen for NanoSIMS imaging. NanoSIMS images reveal specimen 299 contains 6 distinct high Mg-bands in both chambers within the calcite that grew in culture (Fig. 1a–d). Five of the six high Mg-bands (Fig. 1a,c) coincide with the ^87^Sr label that marks the night period (Fig. 1b,d). On the fifth night, the specimen was not transferred into ^87^Sr-labelled seawater; therefore, calcite lacks an ^87^Sr-label (see ‘X' in Fig. 1a–d). Lower Mg/Ca ratio calcite precipitated during the 12 h light periods when the foraminifer was in unlabelled seawater.

Specimen 301 contains four distinct high Mg-bands in the F and F1 chambers that grew in culture (Fig. 1e–h). Similar to specimen 299, the high Mg/Ca calcite was added during the night and coincides with the ^87^Sr-labelled calcite. Although this specimen was transferred five times during the 6 days the foraminifer remained in culture (Supplementary Table 1), we only observe four distinct ^87^Sr-labelled bands in the F and F1 chambers (Fig. 1f,h). During the first two night transfers, multiple thin Mg-bands formed during a single night period (Fig. 1e,g). The calcite that formed during the day between the first and second night transfers contains low-amplitude Mg variability.

### NanoSIMS line profiles from transferred specimens

**Figure 2:**
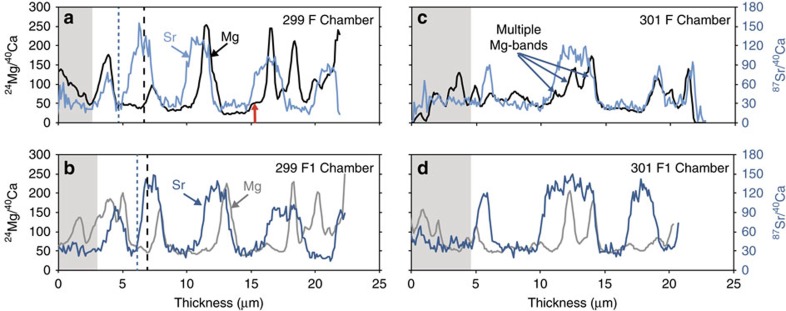
^24^Mg/^40^Ca and ^87^Sr/^40^Ca line profiles are used to assess the timing of specimen transfer into ^87^Sr-labelled seawater and the corresponding Mg-variability. NanoSIMS line profiles for Specimens 299 (**a**,**b**) and 301 (**c**,**d**) were generated along a line perpendicular to the chamber growth direction (white boxes in Fig. 1). The grey boxes denote calcite that formed before the first night transfer into ^87^Sr-labelled seawater. Specimen 299 (**a**,**b**): comparison of the ^87^Sr/^40^Ca (light/dark blue lines) and ^24^Mg/^40^Ca (black/grey lines) for the F (**a**) and F1 (**b**) chambers. Specimen 301 (**c**,**d**): comparison of the ^87^Sr/^40^Ca and ^24^Mg/^40^Ca for the F (**c**) and F1 (**d**) chambers. The dashed lines (**a**,**b**) illustrate the offset between the onset of night calcification (^87^Sr/^24^Ca ratios increase; dashed blue lines) and timing of the increase in the ^24^Mg/^40^Ca ratio (black dashed lines). The red arrow highlights an example of an increase in the Mg/Ca ratio that is synchronous with the ^87^Sr-label in timing, but not magnitude.

NanoSIMS line profiles from the transfer experiment specimens were used to assess the timing of specimen transfer into and out of the ^87^Sr-labelled seawater and the corresponding Mg-variability in a shell wall. These NanoSIMS line profiles confirm that the high Mg-bands in *N. dutertrei* form during the night (Fig. 2), a pattern that is consistent with the timing of Mg-banding reported in the spinose foraminifera *O. universa*^15^. Interestingly, the increase in the Mg/Ca ratio begins after ^87^Sr increases, indicating high Mg/Ca-calcite forms sometime after night calcification begins (see dashed lines on Fig. 2a,b). In addition, the high Mg-bands that form at night are thinner than the ^87^Sr-labelled calcite, which marks the 12 h night cycle. In general, the ^87^Sr-labelled night calcite is thicker than the day calcite that has ambient ^87^Sr/^88^Sr ratios. In some cases, there is a slight increase in the chamber ^24^Mg/^40^Ca that is synchronous with the initial ^87^Sr-label increase at the light to dark transition (for example, Fig. 2a, red arrow). In addition, we observe several distinct high Mg-bands within a single night period (Fig. 2e,f). It is possible that they represent calcite that formed during consecutive night cycles without day calcite precipitation in between. Alternatively, they may be due to variability in the physiological mechanism responsible for Mg-banding in this species. At this time, we cannot explain the occurrence of multiple closely spaced Mg-bands that appear to form during a single night.

### NanoSIMS imaging of control specimens

We observe Mg-banding in both the ocean grown and culture grown (^87^Sr-labelled) calcite in the ‘control' specimens that completed their life cycle in the laboratory, but did not undergo any day/night transfers (Supplementary Figs 4–9). Mg-bands in these specimens are synchronous from chamber-to-chamber (across chamber sutures), indicating each Mg-band formed contemporaneously on all chambers. The observation that the number of Mg-bands in the culture-grown calcite coincides with the number of days these specimens were in culture rules out the possibility that banding in the transferred specimens is due solely to the transfer process itself.

## Discussion

**Figure 3:**
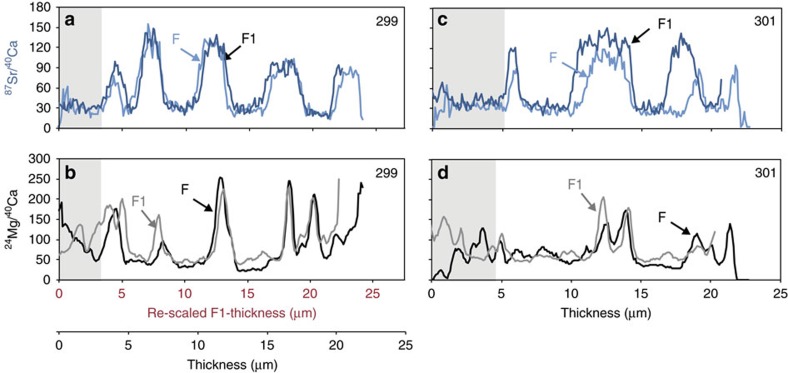
Coherency of Mg-banding between F and F1 chambers in *N. dutertrei* specimens shown with ^24^Mg/^40^Ca and ^87^Sr/^40^Ca line profiles from NanoSIMS images. NanoSIMS line profiles for Specimens 299 (**a**,**b**) and 301 (**c**,**d**) were generated along a line perpendicular to the chamber growth direction (white boxes in Fig. 1). The grey boxes denote calcite that formed before the first night transfer into ^87^Sr-labelled seawater. The ^87^Sr/^40^Ca profiles (light/dark blue lines) are aligned in the upper panels for the Specimens 299 (**a**) and 301 (**c**), which shows the coherency of the timing of the ^87^Sr-label in both chambers. The ^24^Mg/^40^Ca profiles (black/grey lines) (**b**,**d**) suggest that the timing of the Mg-banding is contemporaneous in both chambers. To compare the timing of the Mg-banding in Specimen 229, the *x* axis of the F1 chamber was re-scaled from 0–25 to 0–27 μm. See Supplementary Fig. 3 for details.

The patterns of calcite growth detailed in the NanoSIMS images and line profiles of *N. dutertrei* do not support the classic model of chamber formation in multi-chambered perforate foraminifers^14^,^15^,^16^. LA-ICP-MS depth profiles and NanoSIMS images from specimens 299 and 301 indicate that all chambers are initially thinly calcified (<5 μm wall thickness) and that >80% of the outer shell wall in the final three chambers precipitated after the final chamber formed and shell thickening (day/night Mg-banded calcification) began (Fig. 1 and Supplementary Fig. 1). As these specimens did not add new chambers in culture, but did add ^87^Sr-labelled calcite containing Mg-bands, the process of chamber addition in *N. dutertrei* is not necessary for Mg-banded calcite layers to precipitate over previously formed chambers. The synchronous F and F1 chamber ^87^Sr/^40^Ca and ^24^Mg/^40^Ca NanoSIMS line profiles show that Mg-bands are precipitated simultaneously on the final (F) and penultimate (F1) chambers (Fig. 3). NanoSIMS images of the control specimens that completed their life cycle in the laboratory provide further evidence that most Mg-bands are added after the final chamber forms and are continuous from chamber-to-chamber (Supplementary Figs 4–9). These specimens provide additional evidence that Mg-bands form after the final chamber calcifies. For example, specimen 152B was ∼8–10 μm thick at the time of collection and did not add new chambers in culture. We observe five high Mg-bands in the calcite grown in culture (identified by the elevated ^87^Sr ratios) that are continuous across chamber sutures and appear in the F, F1, F2 and juvenile chambers (Supplementary Figs 4 and 5). One of the most compelling observations comes from an image of the suture between a juvenile chamber and the final chamber in specimen 152B (Supplementary Fig. 5g(1–3)). All of the high Mg-bands span the suture between the juvenile chamber and the F chamber, which lends support to the hypothesis that Mg-banding begins after the F chamber forms. If Mg-bands formed during each chamber addition, then the juvenile chamber would have more Mg-bands compared with the F chamber and not all Mg-bands would be continuous across the juvenile and F chamber sutures.

Control specimens also reveal that very little calcite is added to the inside of the shell during ontogeny despite the considerable amount of calcite added to the outside of the shells after transfer into culture conditions. For example, the F/F1 suture in specimen 152B has one thin Mg-band along the inner chamber surface that is present in both chambers, but is not continuous across the chamber suture; rather it is continuous around the chamber septum (marked with arrows in Supplementary Figs 4 and 5e,h). We postulate that this thin Mg-band formed when the F chamber calcified, and that the F chamber formed during the night when high-Mg calcite is precipitated. Chambers that form during the day, when low Mg-calcite is precipitated, would not have a thin band of high Mg-calcite in the inner most portion of the shell wall (for example, Suture ‘G', Supplementary Fig. 4).

**Figure 4:**
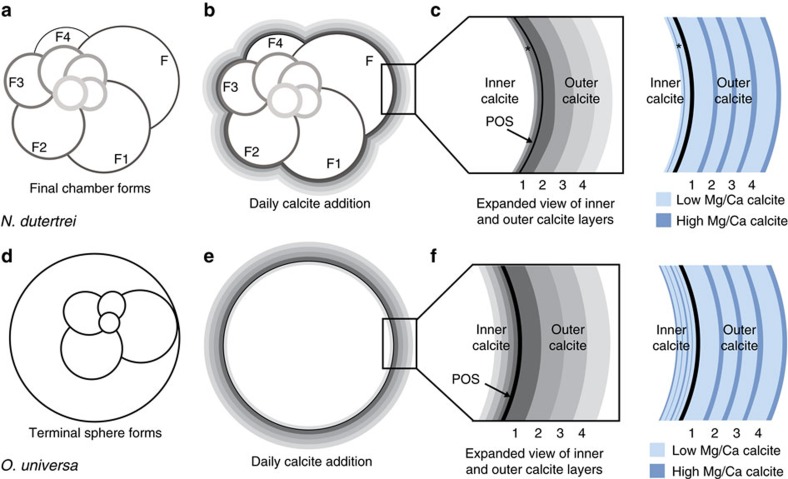
Schematic of proposed chamber growth and thickening for *N. dutertrei* and *O. universa.* In *N. dutertrei*, (**a**) chambers are relatively thinly calcified until after the final chamber forms. (**b**) After the final chamber forms, calcite is added to the outer surface of all chambers during subsequent day/night cycles, with lower Mg calcite precipitated during the day and higher Mg calcite precipitated at night. (**c**) Expanded schematic details daily calcite growth layering and corresponding Mg/Ca variability. The asterisk in **c** marks the location of inner calcite banding in *N. dutertrei*, which is either absent or discontinuously precipitated on the inner chamber walls. In *O. universa*, (**d**) the terminal adult sphere forms. (**e**) Calcite is added daily to the inner and outer surfaces of the shell wall daily. The inner calcite is thinly calcified, whereas the outer calcite is thicker. (**f**) Expanded schematic of the inner and outer calcite layers details corresponding growth on both sides of the POS. Symmetric Mg-bands form on either side of the POS; higher Mg calcite forms during the night.

Based on these observations, we propose a modified model of chamber growth and shell calcification for *N. dutertrei* (Fig. 4). In this model, when *N. dutertrei* adds a new chamber, a very thin veneer of calcite is added to the outside of the previously formed chamber. This process continues until the final chamber forms (Fig. 4a). After the final chamber forms, calcification continues and the foraminifer thickens on a day/night cycle whereby low Mg/Ca calcite forms during the day and high Mg/Ca calcite forms at night (Fig. 4b,c). The identification of synchronous diurnal Mg-bands (Fig. 3) and the continuity of Mg-banding across chamber sutures (Supplementary Figs 4–9) add support to this model and the resulting Mg variability (Fig. 4). It is possible that a thin veneer of calcite is added to the entire shell during chamber formation or that calcification on the previous chambers is sporadic or incongruently added to the shell, which has been observed in the benthic foraminifer *Ammonia aomoriensis*^31^. However, significant precipitation of calcite on previously formed chambers is likely minimal in *N. dutertrei*. If a significant layer of calcite were added over older chambers when a new chamber is formed, the older chambers would be more thickly calcified than the younger chambers, which is not observed in cultured specimens of this species. It is notweworthy that, as discussed in detail below, in specimens that completed their life cycle in the ocean (as opposed to the cultured specimens discussed here), chambers that formed during early ontogeny are often thicker than chambers formed during late ontogeny due to the variability in the thickness of the calcite crust that forms on the outer surface of this species.

Diurnal Mg/Ca banding cycles were reported previously in the spinose foraminifer *O. universa*^15^ and we propose that a slightly modified version of the *N. dutertrei* model can be applied to *O. universa*. Unlike *N. dutertrei*, *O. universa* produces a ‘terminal' calcite sphere that surrounds a juvenile trochospiral shell (Fig. 4d). We suggest that this spherical terminal chamber is analogous to the final chamber in multi-chambered species. Thus, after the final chamber forms, calcification commences on a day/night cycle whereby low Mg/Ca calcite is precipitated during the day and high Mg/Ca calcite is precipitated at night (Fig. 4e). The Mg-banding that would develop based upon this proposed model of calcite growth (Fig. 4f) is supported by day/night labelling experiment results reported for *O. universa*^15^. A key difference between *O. universa* and *N. dutertrei* is that in *O. universa*, the alternating layers of high and low Mg/Ca calcite form contemporaneously on both sides of the POS (Fig. 4e,f), whereas calcification on the inside of the *N. dutertrei* shell is minimal and generally does not contain Mg-banding as in *O. universa* (Supplementary Figs 4–9). Control specimen 152A is the only specimen that displayed inner chamber Mg-banding and it is restricted to the suture between the F and F1 chamber (Supplementary Fig. 7b(1–2)). The inner Mg-bands are neither continuous around the chamber septum between the F and F1 chambers (Supplementary Fig. 7c(1–3)) nor do they extend into the inner calcite of the F chamber (Supplementary Fig. 7a(1–3)). NanoSIMS and/or electron microprobe imaging of other multi-chambered planktic foraminifers show that species with sutural apertures also lack distinct inner calcite Mg-banding or alternatively, that the Mg-bands are thinner than the resolution of the imaging technique (for example, *Pulleniatina obliquiloculata*^32^, *Globorotalia tumida* and *Globorotalia scitula*^33^, and *Globigerinoides ruber*^34^). We suggest that continuous Mg-banding on the inside of the POS is unique to *O. universa* and may be due to the fact that the final spherical chamber in this species is the only example of a final chamber that encompasses all previously formed chambers in the whorl^35^.

The model of chamber formation and ontogenic day/night Mg-banding proposed above might also explain Mg-banding patterns observed in the planktic foraminifer *Neogloquadrina pachyderma*^36^. Jonkers *et al*.^36^ postulate that Mg-banding in *N. pachyderma* is attributed to the chamber formation process which gives rise to the lamellar calcite patterns often observed in *N. pachyderma*. Close inspection of an F chamber from their study (Fig. 3 in Jonkers *et al*.^36^) show multiple Mg-bands within the F chamber that formed before the development of the crust and apertural lip. If Mg-banding were due to chamber formation only, then we would expect one high Mg-band in the F chamber. The number of Mg-bands in *N. pachyderma*, however, are variable from chamber-to-chamber and older chambers appear to have more Mg-bands than the younger chambers (for example, compare Figs 2e,f and 3e,f in Jonkers *et al*.^36^); thus, it is possible that chamber addition and calcification in this species follows the growth patterns described by the classic model^14^. This suggests that foraminifer species may have different or varying modes of chamber formation processes. Culture experiments are likely necessary to confirm ontogenetic growth patterns in this species.

Chamber thickness in individual specimens of *N. dutertrei* varies from chamber to chamber in specimens that completed their life cycle in the natural setting^37^,^38^. Given our observation that all chambers are thinly calcified until the final chamber forms and Mg-banded calcite is added to the entire shell during ontogeny, variable chamber thickness cannot be explained by the model of calcification we propose without considering crust formation. Many non-spinose species including *N. dutertrei*, *N. pachyderma*, *Neogloboquadrina incompta*, *G. scitula*, *Globorotalia menardii*, *Globorotalia truncatulinoides*, *Globorotalia hirsuta* and *Globoconella inflata* form a calcite crust of variable thickness on the outside surface of the ontogenetic Mg-banded calcite^33^,^37^,^39^,^40^,^41^. The calcite crust is often thickest on older chambers (for example, earlier chambers in the shell whorl) and is thin or absent on the younger chambers, giving rise to the variable chamber thicknesses often observed in these foraminifers^37^. Variable crust thickness is common to *N. dutertrei* (Supplementary Fig. 10) and is likely to be responsible for the chamber-to-chamber Mg/Ca ratio differences reported for this species^38^. In specimens with little to no crust, we propose that the Mg/Ca ratios are elevated due the absence of a low Mg/Ca crust (for example, Supplementary Fig. 10a). The crust is often more thickly calcified on the smaller chambers that form during early ontogeny compared with the larger/younger chambers added at the end of shell ontogeny (for example, Supplementary Fig. 10b,c). The crust, which has lower Mg/Ca ratios compared to the ontogenetic calcite, decreases the average Mg/Ca ratio of the crusted chambers^37^,^38^. Therefore, heavily crusted specimens yield lower Mg/Ca ratios compared with shells with little or no crust (for example, Supplementary Fig. 10d).

There are probable fundamental differences between calcification mechanisms controlling crust formation and those responsible for day/night ontogenetic calcification, because crust calcite often has lower Mg/Ca ratios and lacks Mg-banding^30^,^33^,^36^,^37^,^42^, and has distinct oxygen isotopic composition compared to ontogenetic calcite^43^. Laboratory experiments with non-spinose foraminifera suggested that crust addition is a function of temperature reduction as foraminifera settle through the water column^44^. We attempted to trigger crust addition in *N. dutertrei* and two other deep dwellers (*G. menardii* and *G. truncatulinoides*) by transferring specimens that were in culture for several days at 16 °C into cooler water (12 °C) for the remainder of their life cycle. These experiments did not trigger crust formation. However, a *N. dutertrei* maintained in culture at constant temperature completed its life cycle in the laboratory and displayed clear evidence of crust addition on the outer chamber surfaces (Supplementary Fig. 11). NanoSIMS images confirm the absence of Mg-bands in the crust (Supplementary Fig. 11c,d) and a low Mg/Ca ratio in the crust despite being deposited at constant temperature. Crust formation at constant calcification temperature has also been observed in cultured specimens of *N. incompta* (C. Davis, personal communication) and in sediment trap specimens of *N. pachyderma*^36^. Clearly, the mechanisms responsible for crust formation and its Mg/Ca geochemistry remain to be resolved. We note that gametogenic calcite that forms on species such as *Globigerinoides sacculifer* and *O. universa* is different than the crusting discussed here, because gametogenic calcite has a different morphological appearance and is added at the start of meiosis, 24 h before gametogenesis^40^,^45^.

**Figure 5:**
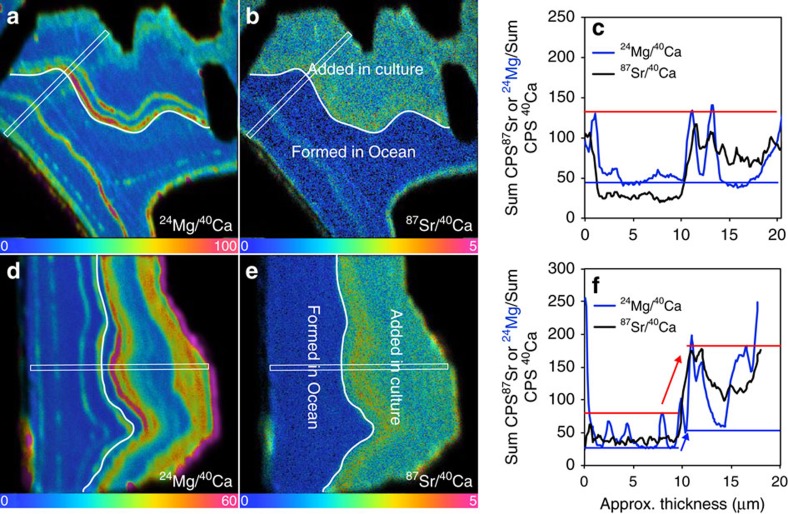
The temperature effect on Mg-banding in two cultured *N. dutertrei* specimens. Temperature effect on Mg-banding in *N. dutertrei* is illustrated by comparing the increase in the Mg/Ca ratios in two specimens collected from the same temperature (12 °C) in the ocean and cultured at (**a**–**c**) 12 °C and (**d**–**f**) 22 °C. (**a**,**b**) NanoSIMS images of the ^24^Mg/^40^Ca and ^87^Sr/^40^Ca of specimen 87 that grew partially in the ocean and then added ∼10 μm of calcite in culture at 12 °C. (**c**) Corresponding line profiles through images (**a**,**b**) (white boxes) reveal the ^24^Mg/^40^Ca ratios do not increase after transfer into cultured water that has the same as the temperature at the depth of collection. (**d**,**e**) NanoSIMS images of the ^24^Mg/^40^Ca and ^87^Sr/^40^Ca of specimen 293 that grew partially in the ocean and then added ∼10 μm of calcite in culture at 22 °C. (**f**) Corresponding line profiles through images (**d**,**e**) reveal the ^24^Mg/^40^Ca of both day and night calcite are higher in the calcite that formed in culture at a temperature 10 °C higher than the temperature at the depth of collection (see arrows). The blue and the red lines denote the lower and upper range of the Mg/Ca ratios, respectively.

The temperature effect on average foraminiferal shell Mg/Ca ratios is well established from empirical calibration studies, demonstrating that multi-shell average Mg/Ca ratios increase with calcification temperature(for example, refs 3, 4). Cultured specimens of *O. universa* show that both the high and low Mg intrashell maxima and minima are modulated by temperature^15^. Assessing differences in intrashell Mg/Ca variability in individual *O. universa* is quite straightforward because the entire terminal sphere grows in culture. In *N. dutertrei*, we illustrate the temperature effect on intrashell Mg/Ca variability by comparing the ocean grown versus culture grown Mg/Ca variability in two specimens that were partially calcified upon collection (temperature at collection depth∼12 °C±1.0 °C) and cultured at different temperatures (12 °C and 22 °C). In the specimen cultured at the ambient temperature of 12 °C, the magnitude of the Mg/Ca ratios of day (low-Mg) or night (high-Mg) calcite precipitated in culture is indistinguishable from calcite precipitated before collection (Fig. 5a–c). In the specimen cultured at 22 °C (10 °C higher than the temperature at the collection depth), cultured calcite has higher Mg/Ca ratios than in the ocean-precipitated calcite (Fig. 5d–f). The *N. dutertrei* Mg/Ca ratios of both day and night calcite bands appear to respond to temperature change as reported for *O. universa*^15^; however, additional analyses are necessary to confirm this finding.

Given the diurnal cyclicity in Mg-banding, it is natural to ask whether symbiotic algae play a role in controlling banding via a shift between photosynthetic and respiration modes on a day/night cycle. There are multiple lines of evidence to suggest symbionts do not trigger Mg-banding. Mg-bands are present in symbiont-barren planktic foraminifers and benthic foraminifers with an infaunal (and thus light limited) habitat (Table 1). This alone suggests symbionts do not play a role in triggering all Mg-banding. However, it may be that in the symbiont-bearing foraminifers, Mg-banding is synchronized with the light cycle due to the presence of symbionts, whereas in symbiont-barren foraminifers Mg-banding is synchronized with some other environmental cue. Another piece of evidence suggesting symbionts do not play a role in triggering Mg-banding is the lag between the timing of transfer of specimens into the dark and the initial rise in chamber Mg/Ca ratios that occurs in both *N. dutertrei* (dashed lines in Fig. 2) and *O. universa*^15^. As the change in extracellular pH associated with symbiont photosynthesis/respiration cycles is nearly instantaneous when lights are turned off/on^46^, the lag in timing between the transfer into the dark and the increase in Mg/Ca ratios suggests symbionts do not trigger an Mg-band to form, as one would expect the foraminifer to respond to the change in pH around the shell instantaneously. However, we do not know whether a change in extracellular pH causes an instantaneous response to the pH within the cytosol of the foraminifers, or how this large pH change affects calcification. If there is a delay in the pH response inside the cytosol or in the response of the foraminifera to a change in extracellular pH, this might explain the timing lag.

Although it appears that symbiosis does not initiate Mg-banding, the diurnal pH change driven by symbiont photosynthesis/respiration cycles may modulate Mg-banding amplitude^15^. Experiments with *O. universa* showed that a pH increase of 0.2 and 0.3 pH units at 20 and 25 °C, respectively, decreased shell Mg/Ca ratios by an average of 34 and 9%, respectively^15^. The authors concluded that the average change in Mg/Ca ratios (34 and 9%) was too small to explain the 200–400% change in the Mg/Ca ratios between day/night calcite measured in individual specimens. Yet, *O. universa* can experience a 1 pH unit range in extracellular pH between light/dark condition^46^. Thus, the diurnal change in pH may yield a much larger change in the average Mg/Ca ratio than the pH change used by Spero *et al*.^15^. The diurnal pH change has not been measured in *N. dutertrei* or asymbiotic species, thus, the complicated interplay between pH, temperature, Mg/Ca ratios and banding amplitude in other multi-chambered species, in particular those without algal symbionts, needs to be explored further.

A number of studies have explored biomineralization mechanisms responsible for the incorporation of trace elements into foraminifera shells over the last two decades^18^,^23^,^31^,^47^,^48^. A Rayleigh fractionation model was proposed to explain trace element variability in benthic foraminifera^47^, but this model is too simplistic to explain the day/night banding evident in most planktic species and it cannot account for compositional variability of some elements^33^. Studies with the hyaline benthic foraminifer *Amphistegina lobifera* suggest seawater is vacuolized via endocytosis, modified to reduce the Mg^2+^ content via ion-transport pumps, alkalized to raise the total carbon, and transported to the site of calcification by the cell^48^. Mg-rich particles inside the cytosol are also evidence of the active removal of Mg during calcification in this genus^49^. Studies with the benthic foraminifer *Cibicides lobatus* confirm the pH in the calcifying fluid is elevated at the site of calcification, suggesting that foraminifer species promote calcification by elevating pH in seawater vacuoles^50^. A subsequent study proposed that seawater vacuolization, termed passive-transport, plays a smaller role in the biomineralization process and suggested a combination of passive transport and transmembrane transport of ions through selective channels to explain elemental content in foraminifera^31^. Importantly, most of these studies investigated shell biomineralization in benthic foraminifer species^51^. There is a clear need to examine biomineralization in planktic species, as chamber formation and chamber calcification processes may not be the same between these groups.

When first identified, Mg heterogeneity in planktic foraminifera prompted questions regarding the validity of the Mg/Ca-temperature proxy and the implications of Mg heterogeneity for paleoceanographic reconstructions needed to be explored^14^,^52^. Changes in calcification temperature due to migration in the water column are not responsible for Mg-banding since the temperatures predicted by the range of Mg/Ca ratios within foraminiferal calcite (1 to >20 mmolper mol Mg/Ca) is far too large (>30 °C, estimated from Mg/Ca-calibration relationships) compared with the temperature range that could be experienced during a typical foraminifer's life cycle (<10 °C). In addition, Mg-bands are present in foraminifers that are cultured at constant temperature (ref. 13 and this study). The presence of Mg-banding in both symbiont-bearing and asymbiotic species indicates photosymbionts are not directly responsible for banding, although the effect of pH change between photosynthesis- and respiration-dominated periods could influence the timing and amplitude of Mg-bands in symbiont-bearing species^3^,^15^. Here, the timing and chamber-to-chamber patterns of Mg-banding in *N. dutertrei* is similar to Mg-banding in *O. universa*^15^. Despite occupying different ecological niches, the timing of Mg/Ca variability in these two species appears to be controlled by changes in the daily light cycle. Very thin Mg-bands may form during chamber addition if the chamber forms during the night when high Mg/Ca ratio calcite is precipitated. This may give rise to very thin high Mg/Ca ratio calcite layers during chamber formation (for example, arrows in Supplementary Fig. 4b3,c3). However, these very thin high Mg-bands are not resolvable without NanoSIMS imaging. Distinct Mg-bands arise from day/night calcification processes during shell thickening and are therefore not directly related to the chamber addition process. These results suggest Mg-banding is an inherent component of biomineralization in planktic foraminifers and is probably linked to common physiological processes that are subsequently modulated by environmental conditions such as temperature, pH and the diurnal light cycle. Experimental studies are necessary to confirm the mechanism for this causal relationship in other planktic foraminifers, especially those without algal symbionts that are often found below the photic zone (for example, *Globigerina calida* and *Globorotaloides hexagona*^53^), species that live in continuous light conditions at high latitudes in the summer (*N. pachyderma* and *Globigerina bulloides*) and in benthic foraminifers including those with infaunal (and thus continuous dark) habitats^54^.

## Methods

### Laboratory culture methods

Live specimens of *N. dutertrei* were collected in July 2014 from a water depth of ∼30 m via an open–close plankton net (Aquatic Research, 150 μm mesh) in the San Pedro Basin, Southern California Bight (33°23'N, 118°26'W) and cultured at the Wrigley Marine Science Center, Santa Catalina Island, California. The temperature at the collection depth, measured with a Sea-bird Electronics SBE19plus profiler, was 16.0 °C±0.1.

Our culture protocol was modified from the method established for non-spinose planktic foraminifer^55^. For these experiments, newly collected specimens were transferred into 70 ml polystyrene Falcon culture flasks containing filtered (0.2 μm nitrate cellulose filters) ambient seawater that was obtained at the collection site. Specimens were maintained in water baths at 16±0.1 °C and illuminated at 55±15 μmol photons per m^2^ s^−2^ using LED XP-E 72 Watt CREE lights (full spectrum) on a 12 h light:12 h dark cycle. Every other day, each foraminifer was fed a 1-day-old previously frozen *Artemia* nauplius by pipetting the thawed *Artemia* onto the specimen's rhizopodial network. If the rhizopodial network was retracted, the foraminifer was not fed. Specimens were maintained in ambient seawater during the 12 h light period (08:00–20:00 h) and then transferred into modified seawater during the dark period (20:00–08:00 h) to label night calcification. We modified the seawater by increasing the ^87^Sr/^88^Sr ratio of the seawater to 4 × the natural isotopic abundance ratio (from ∼0.084 to ∼0.34). Control specimens were placed in ^87^Sr-labelled seawater after collection to label culture grown calcite. They remained in the ^87^Sr-labelled seawater until the end of their life cycle and did not undergo any night transfers. We used ^87^Sr to label the calcite by increasing the ^87^Sr/^88^Sr ratio of the seawater to 4 × the natural isotopic abundance ratio (from ∼0.084 to ∼0.34). The ^87^Sr/^88^Sr ratio of natural (0.086±0.002, *N*=2) and spiked seawaters (0.344±0.007, *N*=14) was determined using an Agilent Technologies 7,500a ICP-MS in the UC Davis Center for Plasma Mass Spectrometry. The ^87^Sr counts were obtained by removing the contribution of ^87^Rb to total counts at mass 87, using ^85^Rb counts and the ^85^Rb/^87^Rb natural abundance ratio (0.3856). The transfer procedure was repeated daily (Supplementary Table 1). At the end of the experiment, the shells were rinsed in deionized water and archived in micropaleontology slides for later analysis.

### Sample cleaning and LA-ICP-MS methodology

Before analysis, individual *N. dutertrei* were cleaned in a 1:1 solution of 30% H_2_O_2_ and 0.1 N NaOH for 10 min at ∼65 °C to remove remnant organics^56^, followed by three rinses in Milli-Q water (18 MΩ). Individual chambers were amputated using a scalpel and mounted on a glass slide prepared with double-sided carbon tape. Specimens were oriented with the inner surface facing up to obtain sub-micron high-resolution data^57^,^58^.

Trace element profiles were obtained using a Teledyne Photon Machines Analyte G2 193 nm excimer laser with a HelEx dual-volume laser ablation cell coupled to an Agilent 7,700x quadrupole-ICP-MS (Supplementary Table 2). Ablated material is transported to the ICP-MS in a He-Ar gas mixture via a ten-path distributed delay manifold that dampens laser pulse harmonics^59^. Gas composition and flow rate (tuned daily) were determined by adjusting the flow of Ar and He to achieve high count rates on the sample/standard, while maintaining ThO^+^/Th^+^ ratios <0.4%. The isotopes ^24^Mg, ^25^Mg, ^27^Al, ^43^Ca, ^44^Ca, ^87^Sr, ^88^Sr and ^138^Ba were measured using a rapid peak hopping procedure. Repeat analyses were obtained on fragments that were large enough to permit multiple laser spots. Following our laboratory protocol, we analysed a single fossil *O. universa* shell repeatedly as a consistency standard to assess depth profile reproducibility within and between analytical sessions^60^. A total of 19 replicate analyses were obtained from the same fossil *O. universa* from October 2013 to June 2015. The mean Mg/Ca ratio from these repeat analyses was 7.1±0.4 mmol mol^−1^ (1*σ*). Element/Ca ratios for the foraminifera were calculated offline in MS Excel, following established data reduction protocols^61^ that included screening for outliers, drift correcting by bracketing samples with NIST SRM 610 analyses and subtracting average background counts (calculated with the laser off) from each data point. The mean element/Ca ratio for each profile is calculated by normalization to the known trace element concentrations in the drift-corrected bracketed analyses of the NIST SRM 610 standard^62^. We used ^43^Ca as our internal standard. We also monitored ^27^Al to indicate ablation through the shell wall into the carbon tape (which yields a diagnostic signal at ^27^Al; see Supplementary Fig. 3 in Fehrenbacher *et al*.^60^).

### NanoSIMS preparation and imaging

After LA-ICP-MS analysis, the shell fragments of two specimens chosen for NanoSIMS analysis (Specimens 299 and 301) were removed from the carbon tape using methanol, embedded in epoxy resin (Struers EpoFix), polished with a series of diamond polishing pastes and then coated with a ∼15–20 nm layer of gold to provide conductivity at high voltage.

NanoSIMS analyses were performed at the Pacific Northwest National Laboratory using a Cameca NanoSIMS 50 l. An 16 keV O^−^ beam was scanned over surface areas ranging in size from 20 × 20 to 30 × 30 μm^2^ depending on the sample. Primary beam current was ∼3 pA. Before analysis, the sample area was pre-sputtered for several minutes to remove Au-coating and surface contaminants. Positive secondary ions (^24^Mg^+^, ^43^Ca^+^, ^40^Ca^+^, ^55^Mn^+^, ^87^Sr^+^ or ^88^Sr^+^ and ^138^Ba^+^) were collected simultaneously in six electron multipliers. Image collection took ∼1–2 h. Each image consisted of 20–40 frames that were then shift corrected (if necessary) and summed to generate a single image. Data were processed using the Open-MIMS plugin (http://www.nrims/hms/harvard.edu) for ImageJ (Ahttp://rsbweb.nih.gov/ij/).

Line profiles were generated across the NanoSIMS images (Fig. 1) to evaluate the timing of Mg-banding in the final (F) and penultimate (F1) chambers of both specimens (Figs 2 and 3). Owing to the orientation of the shell fragments during embedding, the precise placement of the line profiles on the NanoSIMS images and/or small differences in shell thickness, the line profiles from Specimen 299 were aligned by slightly modifying the *x* axis of the F1 line profile. As the ^87^Sr-label should be synchronous in both chambers, we used the ^87^Sr/^40^Ca line profiles to align the line profiles (see Supplementary Fig. 3).

### Data availability

The data generated during this study are available from the corresponding author upon reasonable request.

## Additional information

**How to cite this article:** Fehrenbacher, J. S. *et al*. Link between light-triggered Mg-banding and chamber formation in the planktic foraminifera *Neogloboquadrina dutertrei*. *Nat. Commun.* **8,** 15441 doi: 10.1038/ncomms15441 (2017).

**Publisher's note:** Springer Nature remains neutral with regard to jurisdictional claims in published maps and institutional affiliations.

## Supplementary Material

Supplementary Information Supplementary Figures, Supplementary Tables and Supplementary Discussion

## Figures and Tables

**Table 1:** Summary of planktic and benthic species with distinct Mg-banding.

**Species**	**Habitat**	**Photosymbionts**
*O. universa*^14^,^15^,^52^,^58^,^63^	Planktic	Yes
*G. ruber*^30^,^34^,^57^,^64^	Planktic	Yes
*G. sacculifer*^57^	Planktic	Yes
*Globigerinoides conglobatus*^64^	Planktic	Yes
*Neogloboquadrina dutertrei*^30^	Planktic	Yes
*P. obliquiloculata*^32^,^57^	Planktic	Yes
*Globorotalia inflata*^33^	Planktic	Yes
*G. menardii*^64^	Planktic	Yes
*G. scitula*^33^	Planktic	No
*N. pachyderma*^36^	Planktic	No
*N. incompta*^42^	Planktic	No
*G. truncatulinoides*^57^	Planktic	No
*A. lobifera*^18^	Benthic	No
*Amphistegina lessonii*^65^	Benthic	Yes
*Uvigerina peregrina*^54^	Benthic	No
