# Supplementary material for: Link between light-triggered Mg-banding and chamber formation in the planktic foraminifera Neogloboquadrina dutertrei
Source: Nat Commun. 2017 May 15;8:15441. doi: 10.1038/ncomms15441 (PMC5440661; doi:10.1038/ncomms15441)
Supplement: Supplementary Information — Supplementary Figures, Supplementary Tables and Supplementary Discussion [file ncomms15441-s1.pdf]

Supplementary Table 1: Culture notes

Specimen	Days in culture	Number of Transfers	Cytoplasm color on collection day	Cytoplasm color at end of culture	Day(s) fed	Additional Notes
266	8	6	Orange	White	1, 3, 5, 8	Cytoplasm was white on day 6. Culled prior to gametogenesis.
299	5	5	Pink	Pink	2, 5	Culled prior to gametogenesis
301	6	6	Red	Pink	2, 5	Culled prior to gametogenesis
304	5	5	White	White	Not fed	Pregametogenic the entire time it was in culture, released gametes on day 5
152A	5	1*	Brown	White	2	Pregametogenic on day 4, empty on day 6
152B	5	1*	Brown	White	2	Pregametogenic on day 4, shells empty on day 6
241	3	1*	Red	White	1	Pale in color on day 2, white on day three.

*Specimens transferred into ^{87}Sr labeled seawater after collection and remained in labeled seawater

Supplementary Table 2: Operating conditions of the laser ablation and ICP-MS differential

**ICPMS: Agilent 7700x**	
RF Power	1350 W
Argon (carrier) gas flow	0.95 – 1.0 l/min (tuned daily)
Ar Coolant gas flow	15 l/min
Ar Auxiliary gas flow	1 l/min
Dwell time per mass	20-50 ms (variable)
Total sweep time	307 ms
**Laser-ablation system: UV Excimer Laser**	
Energy density (fluence)	1.0-1.5 J/cm^2 (variable)
He gas flow	1.05 l/min
Laser repetition rate	4-5 Hz
Laser spot size	40-50 μm
ThO^+/Th^+	< 0.4%

Supplementary Discussion:

LA-ICP-MS results of specimens exposed nightly to ^{87}Sr label

$^{87}Sr/^{88}Sr$ ratios (blue lines, Supplementary Fig. 1) were used to distinguish between calcite that precipitated in the ocean and calcite formed under culture conditions during day and night periods. Calcite that grew in the ocean and prior to the first night transfer into ^{87}Sr-labeled seawater (grey boxes, Supplementary Fig. 1) had ambient $^{87}Sr/^{88}Sr$ ratios (~0.084). At collection, these specimens were thinly calcified (chamber walls were <5 μm thick) and had a newly formed final chamber (Supplementary Table 1, Supplementary Figs 1a-f). Laser spot analyses revealed variable Mg/Ca ratios and evidence of Mg-banding in the cultured calcite. Several high Mg-bands that formed in culture were enriched in ^{87}Sr indicating that they formed at night. The number of high Mg-bands is generally consistent with the number of day/night cycles the specimens experienced in the laboratory (Supplementary Table 1).

NanoSIMS imaging (following section) reveal that thin Mg-bands that are closely spaced or 'wavy' in nature are not always resolvable using LA-ICP-MS, which is expected given the nature of how the two types of profiles are generated. For NanoSIMS, an approximate 1.5μm region of a 2D NanoSIMS image is integrated (data is integrated perpendicular to the line profile) to generate the line profiles (e.g. white boxes in Figure 2). In contrast, the laser ablation profiles are generated using a 30-50 μm round or square laser spot (~700 to 2000 micron area) that pulses through the shell starting from the flat inner shell surface to the outer shell surface, thus, the laser depth profiles integrate a larger amount of data per pulse compared to the nanoSIMS line profiles. Owing to the nature of the Mg-bands in *N. dutertrei*, the integrated data using the laser may contain a mixture of high and low Mg/Ca calcite, which yields a mixed signal. This is due, in part, to the nature of how the Mg/Ca bands form in *N. dutertrei*. Initially, Mg-bands in *N. dutertrei* form parallel to the inner shell wall, but they become 'wavier' towards the outer portion of the shell wall. The 'waviness' of the outer Mg-bands is correlated with the surface topography of the shell. As the laser ablates through the shell, it first encounters trace element bands that are perpendicular to the laser beam (and thus Mg-bands are easily resolvable). As ablation progresses the beam encounters wavy Mg-bands and, therefore, a mixture of low and high Mg/Ca calcite. Because both high and low Mg/Ca calcite is ablated at the same time, the amplitude of the Mg-banding is reduced in the resulting depth profile. Additionally, as the laser penetrates through the shell wall, the beam can interact with the sidewall of the spot being ablated, which can further obscure, or mix, the trace element signal. Laser ablation profiles are, therefore, initially higher-resolution at the beginning of the depth profile with abrupt transitions between trace element banding. As ablation progresses, the combination of the wavier trace element bands and any sidewall interactions yield a more mixed signal and reduced amplitude

banding with smoothed transitions between trace element variations. We refer to this as an 'analytically mixed' signal.

We simulate the effects of a laser-based 'mixed signal' by generating two line profiles (integrated perpendicular to the line profile) through nanoSIMS image from Specimen 299. The first profile is generated using a 1.5-μm portion of the intensity image, which yields high amplitude Mg-bands in the line profile (blue lines, Supplementary Figs 1e,f). In contrast, a 24-μm thick line profile yields line profiles that have diminished trace element banding because the integrated regions contain a mixture of both high and low Mg/Ca calcite (red lines, Supplementary Figures 1e,f). Laser profiles integrate an even larger area and therefore may yield even lower amplitude, or obscured, banding.

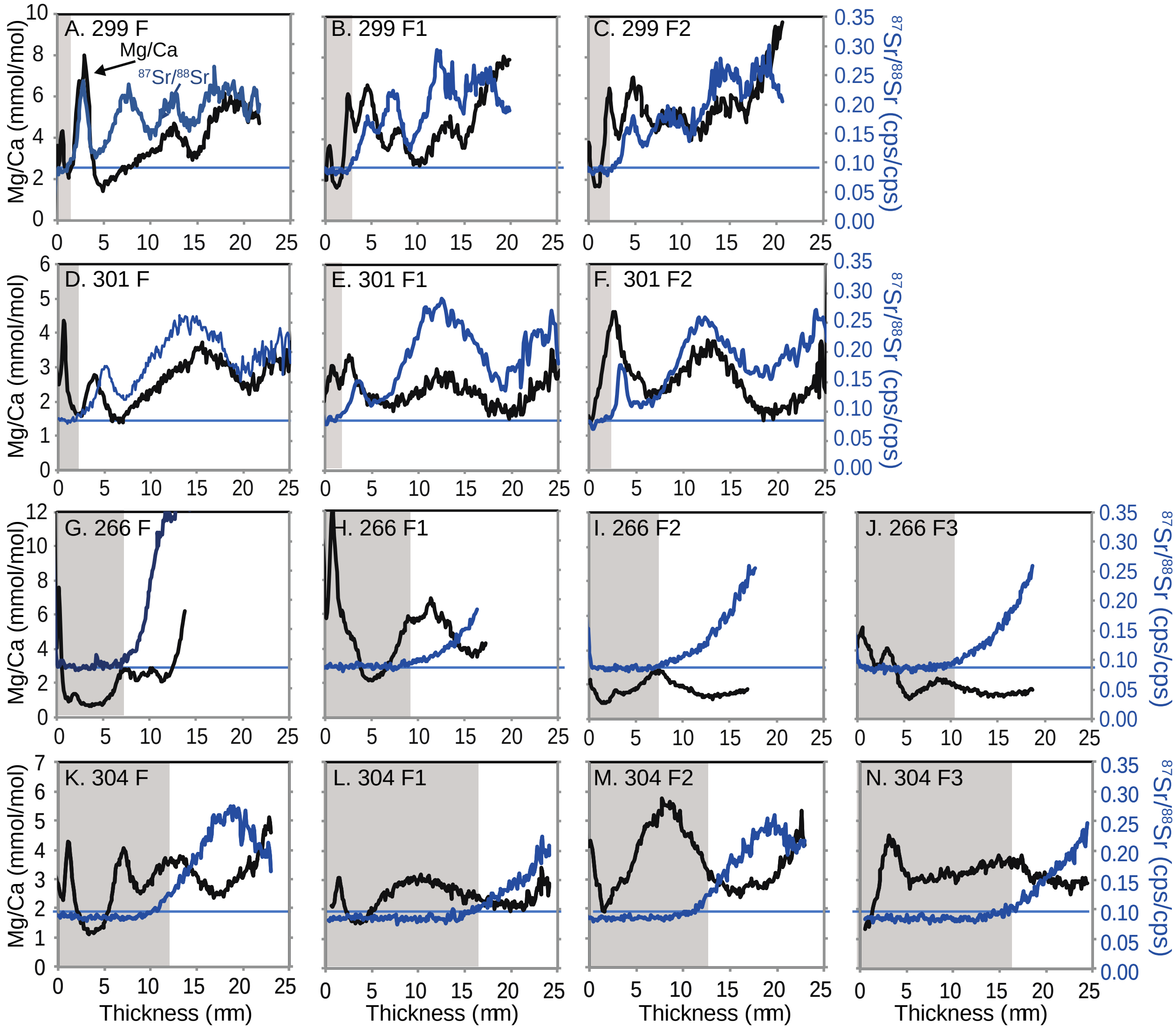

Supplementary Figure 1. Laser ablation profiles of the Mg/Ca (in mmol/mol; black line) and $^{87}Sr/^{88}Sr$ (in cps/cps; blue line) for the F through F2 or F3 chambers.
Shells, ablated from the inner to the outer surface, are used to assess culture results and choose specimens for NanoSIMS analysis. Calcite that grew prior to collection and the first night transfer into ^{87}Sr-labeled seawater (grey boxes) is identified using ambient $^{87}Sr/^{88}Sr$ ratios (horizontal blue line marks the ambient $^{87}Sr/^{88}Sr$ ratio; ~0.084). (A-C) Specimen 299. (D-F) Specimen 301. (G-J) Specimen 266. (K-N) Specimen 304.

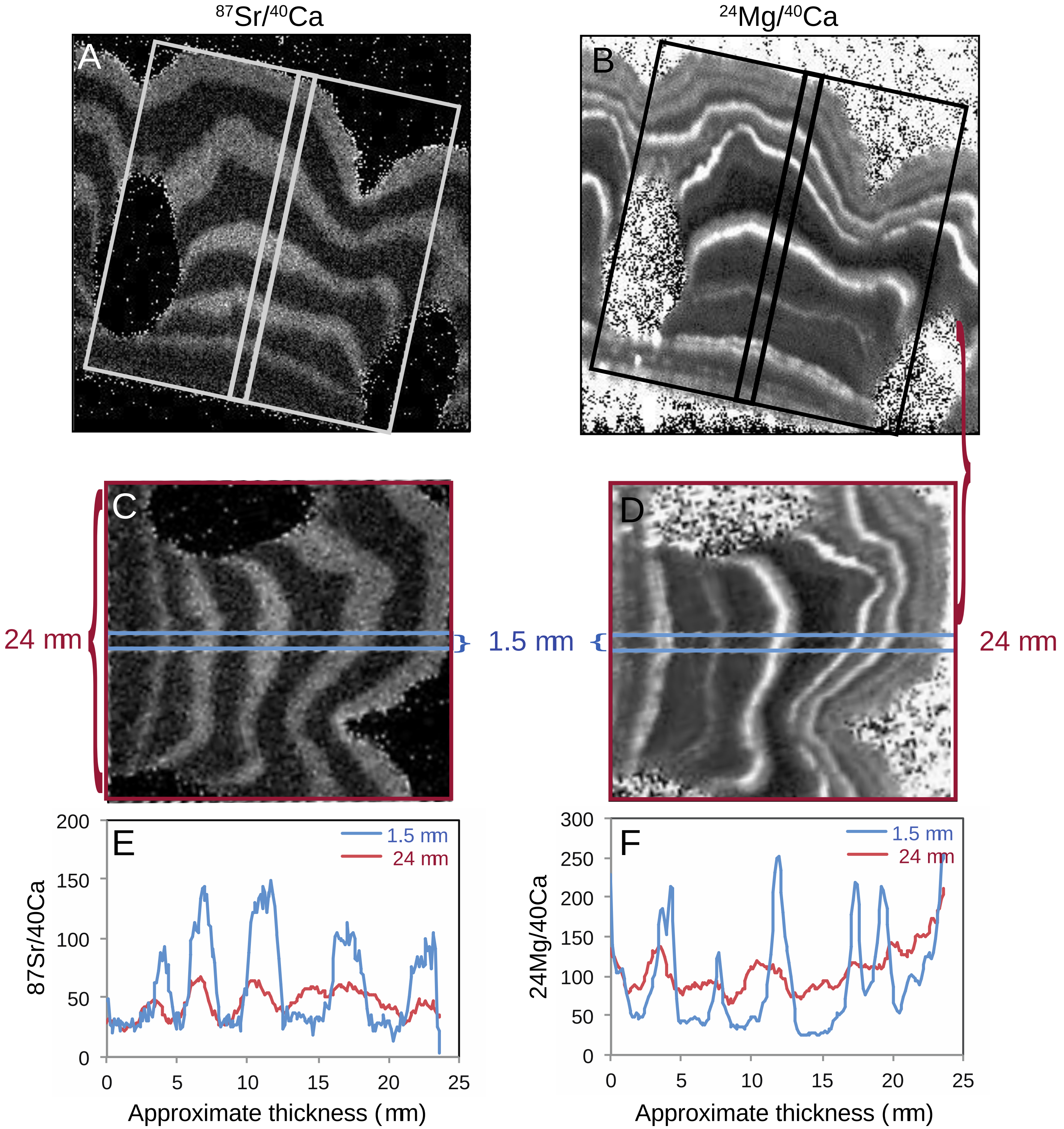

Supplementary Figure 2. Line profile comparison.

Line profiles generated through (A) the $^{87}Sr/^{40}Ca$ and (B) $^{24}Mg/^{40}Ca$ nanoSIMS intensity image maps were generated by integrating 1.5 µm (blue boxes and corresponding blue lines) and 24 µm (red boxes and corresponding red lines) thick portions of the data. The areas used to generate the line profiles are rotated (C and D) so that the line profiles (E and F) can be correlated more readily with the NanoSIMS images. The line profiles generated using 1.5 µm regions yield abrupt trace element transitions. The line profiles generated using 24 µm regions yield smoothed line profiles with reduced amplitude because both high and low Mg/Ca ratio calcite is integrated together.

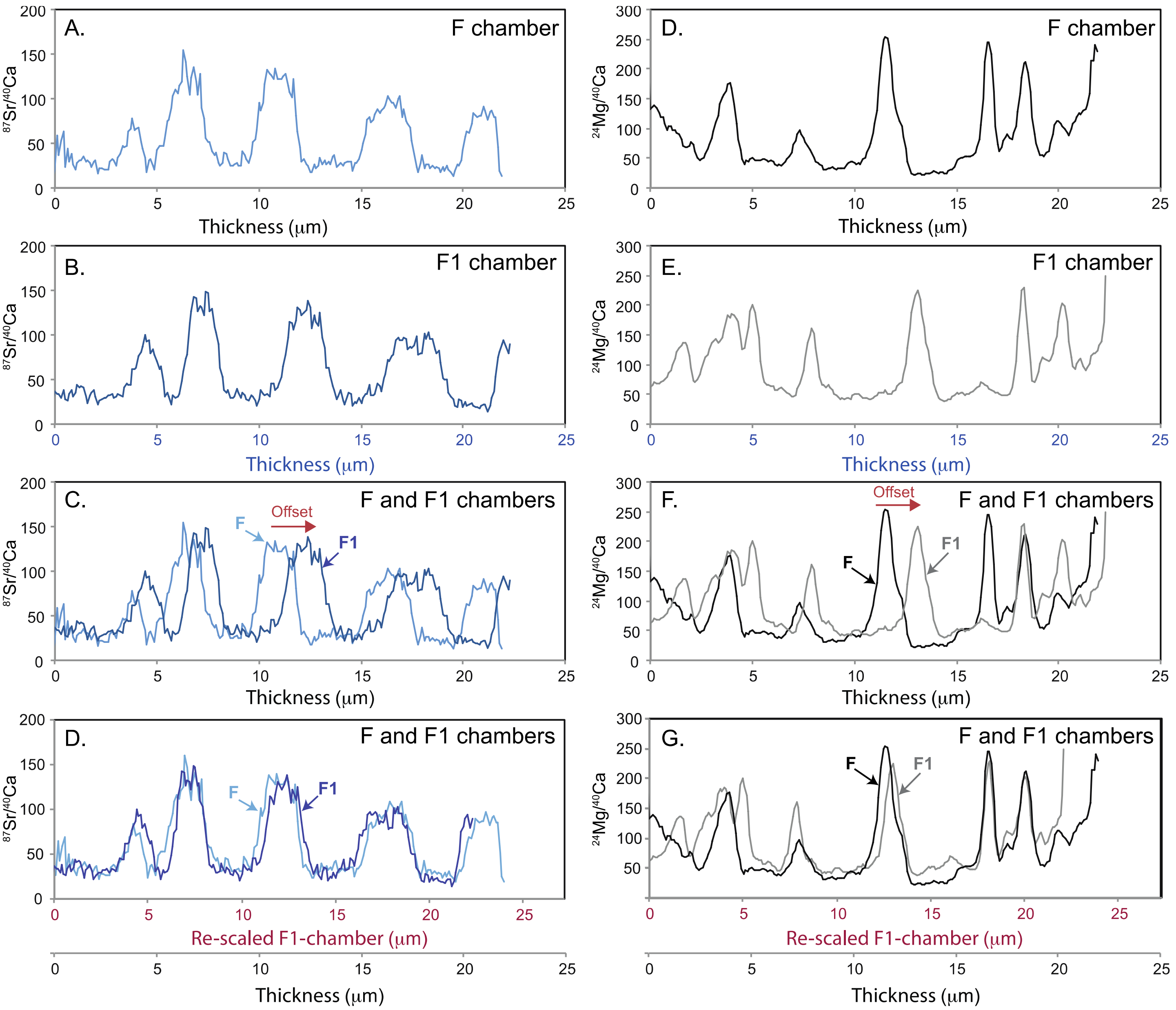

Supplementary Figure 3. Procedure for aligning the ^{87}Sr/^{40}Ca and ^{24}Mg/^{40}Ca line profiles from for the F and F1 chambers of Specimens 299.

The x-axis for the F1 chamber in Specimen 299 was expanded by 2 μm to align the ^{87}Sr/^{40}Ca bands between chambers. We use the ^{87}Sr/Ca ratio to align the data because the ^{87}Sr-spike is contemporaneous in both chambers. We then align the ^{24}Mg/^{40}Ca line profiles from the F and F1 chambers using the same 2 μm shift used for the ^{87}Sr/^{40}Ca alignment. Specimen 301 did not require an x-axis realignment. (A) F chamber ^{87}Sr/^{40}Ca line profile. (B) F1 chamber ^{87}Sr/^{40}Ca line profile. (C) the ^{87}Sr/^{40}Ca line profiles of both the F and F chamber prior to F1 x-axis realignment. (D) ^{87}Sr/^{40}Ca data for both chambers after x-axis for F1 was rescaled from 0-25 μm to 0-27 μm. (E-G) ^{24}Mg/^{40}Ca data from the F and F1 chambers. (H) ^{24}Mg/^{40}Ca data after x-axis for F1 was rescaled from 0-25 μm to 0-27 μm.

Supplementary Figures 4-9: SEM and/or NanoSIMS images of *N. dutertrei* specimens that completed their life cycle in the laboratory. Unless otherwise noted, NanoSIMS data collection included ^{24}Mg, ^{40}Ca, ^{87}Sr, and/or ^{88}Sr.

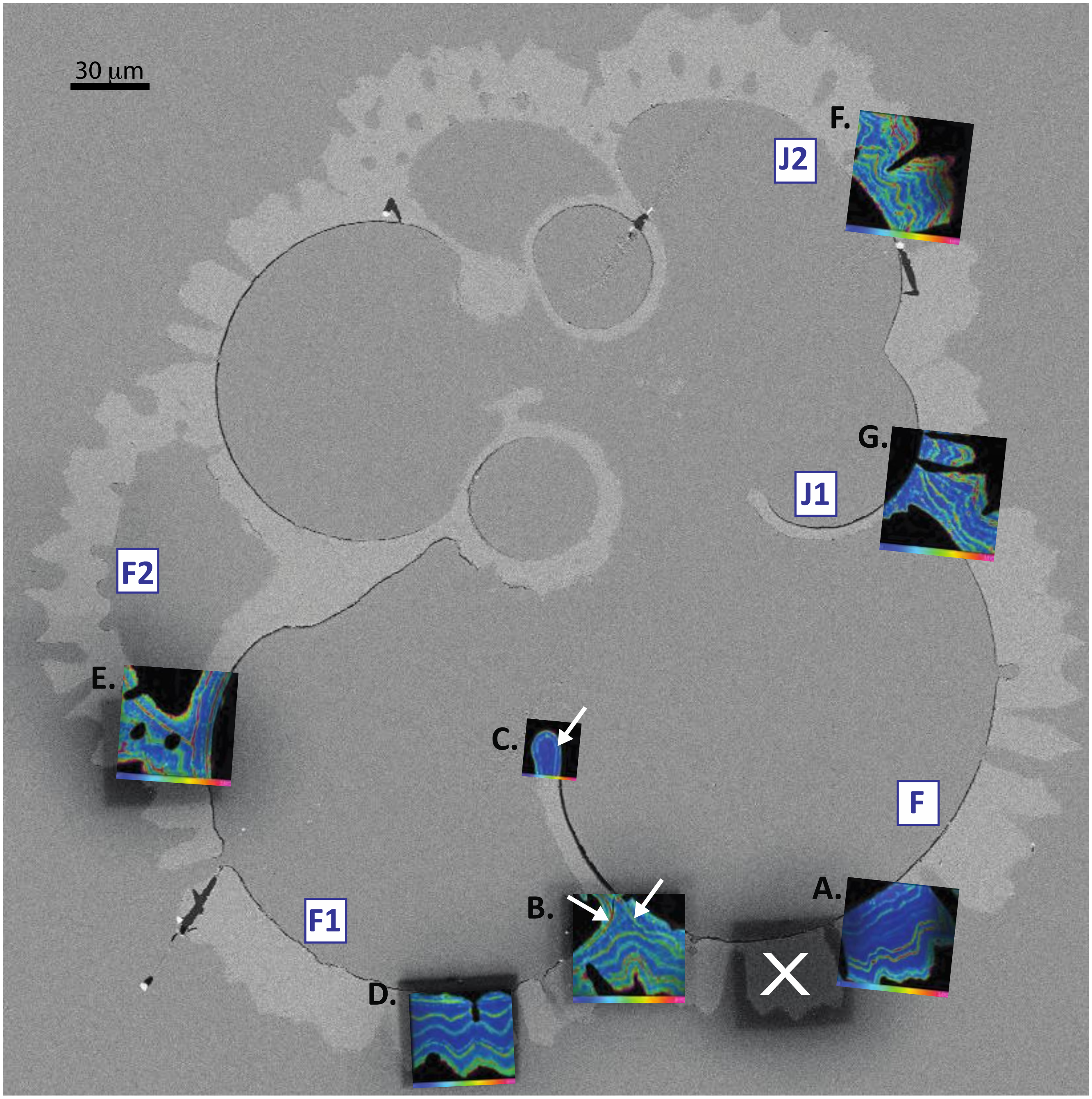

Supplementary Figure 4. SEM of a polished cross-section of specimen 152B
^{24}Mg/^{40}Ca NanoSIMS images are superimposed to identify the locations of the enlarged NanoSIMS images detailed in Supplementary Fig. 5. See Supplementary Fig. 5 for enlarged images. Note: warm colors = higher ratios. The 'X' in the image below marks the location of a NanoSIMS image that was not completed. These images represent the summation of a stack of 20-40 NanoSIMS frames that were shift-corrected; the hue scale bar represents the ratio of the summed ^{24}Mg, ^{87}Sr, and/or ^{88}Sr counts divided by ^{40}Ca counts × 10000.

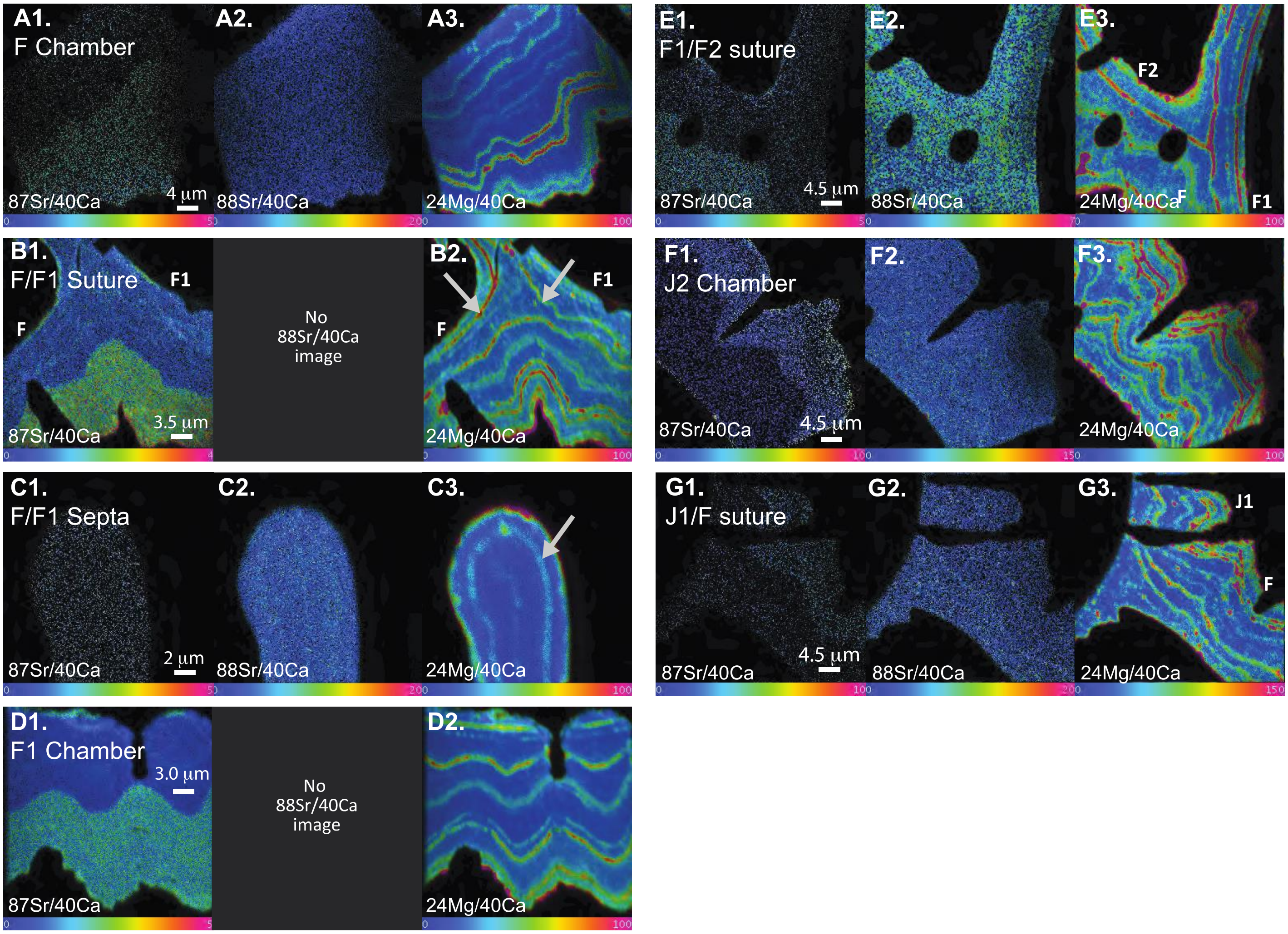

Supplementary Figure 5. Intensity ratio maps of $^{87}Sr/^{40}Ca$, $^{88}Sr/^{40}Ca$, and $^{24}Mg/^{40}Ca$ for specimen 152B. This specimen was less than 10 µm thick at the time of collection and the thickness of the chamber wall was increased by 10 µm while in culture. Image letters correspond to the superimposed images on the previous page: (A) F chamber. (B) Suture between F and F1 chambers. (C) Septa between F and F1 chambers. (D) F1 chamber. (E) Suture between F1 and F2 chambers. (F) Juvenile chamber (J2 from Supplementary Fig. 4). (G) Suture between a juvenile chamber (J1 from Supplementary Fig. 4) and the F chamber.

Note: Gray arrows in B2 and C3 mark the location of an Mg-band that likely formed when the F chamber calcified. It appears continuous from the inside of the F chamber, around the septum, and into the F1 chamber.

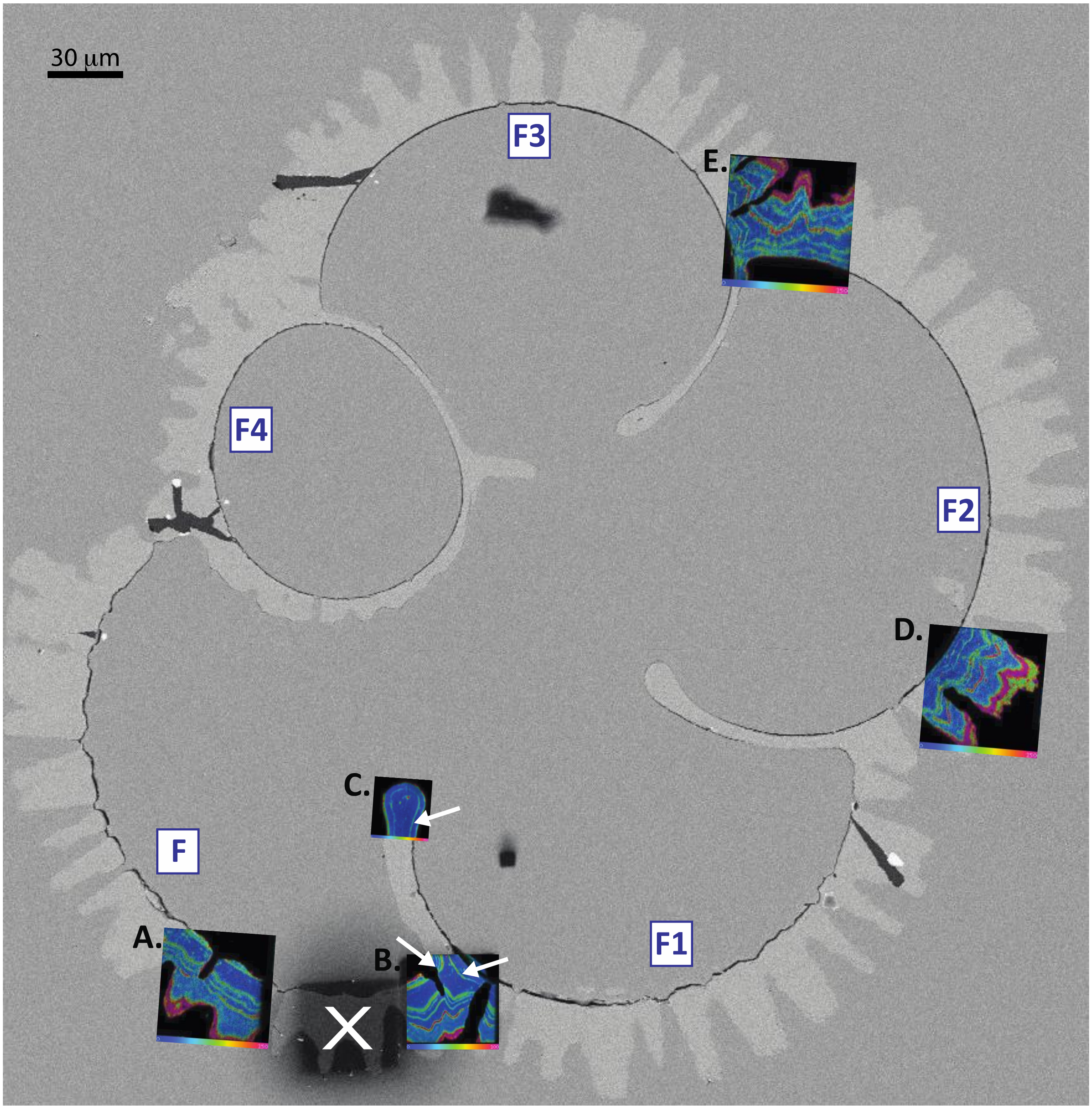

Supplementary Figure 6. SEM of a polished cross-section of specimen 152A.
$^{24}Mg/^{40}Ca$ NanoSIMS images are superimposed to identify the locations of the enlarged NanoSIMS images detailed in Supplementary Fig. 7. White arrows in B and C denote an Mg-band that is continuous from the F, around the septum, and extends into the F1 chamber. See Supplementary Fig. 7 for enlarged figures. Note: warm colors = higher ratios. The 'X' in the image below marks the location of a NanoSIMS image that was not completed. These images represent the summation of a stack of 20-40 NanoSIMS frames that were shift-corrected; the hue scale bar represents the ratio of the summed ^{24}Mg, ^{87}Sr, and/or ^{88}Sr counts divided by ^{40}Ca counts × 10000.

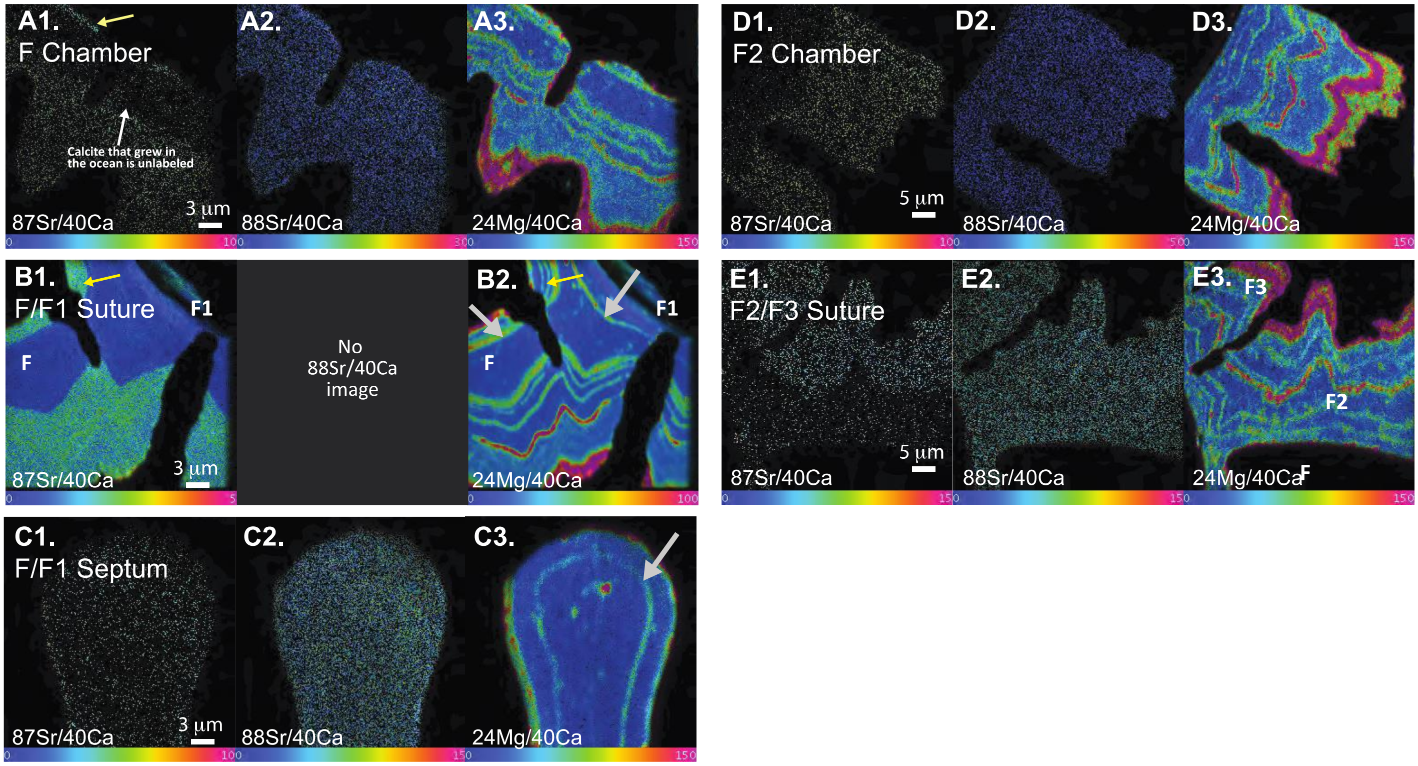

Supplementary Figure 7. Intensity ratio maps of $^{87}Sr/^{40}Ca$, $^{88}Sr/^{40}Ca$, and $^{24}Mg/^{40}Ca$ for specimen 152A. This specimen was less than 5-7 µm thick at the time of collection and added approximately 10 µm of calcite in culture. Image letters correspond to the superimposed images on the previous page: (A) F chamber. (B) Suture between F and F1 chambers. (C) Septa between F and F1 chambers. (D) F2 chamber. (E) Suture between F2 and F3 chambers.

Note: Gray arrows in B2 and C3 mark the location of the Mg-band that likely formed when the F chamber calcified. It appears continuous from the inside of the F chamber, around the septum, and into the F1 chamber. All other Mg-bands are continuous around all chambers. The yellow arrows in figures A1, B1 and B2 are evidence of a small amount of calcite precipitated onto the inside of the shell wall. It is thickest inside the chamber suture (B1, B2) and is very thin in the F chamber suggesting this layer is not continuous around the entire inner shell wall. It is absent inside the F2 and F3 chambers where no inner calcite formed in culture.

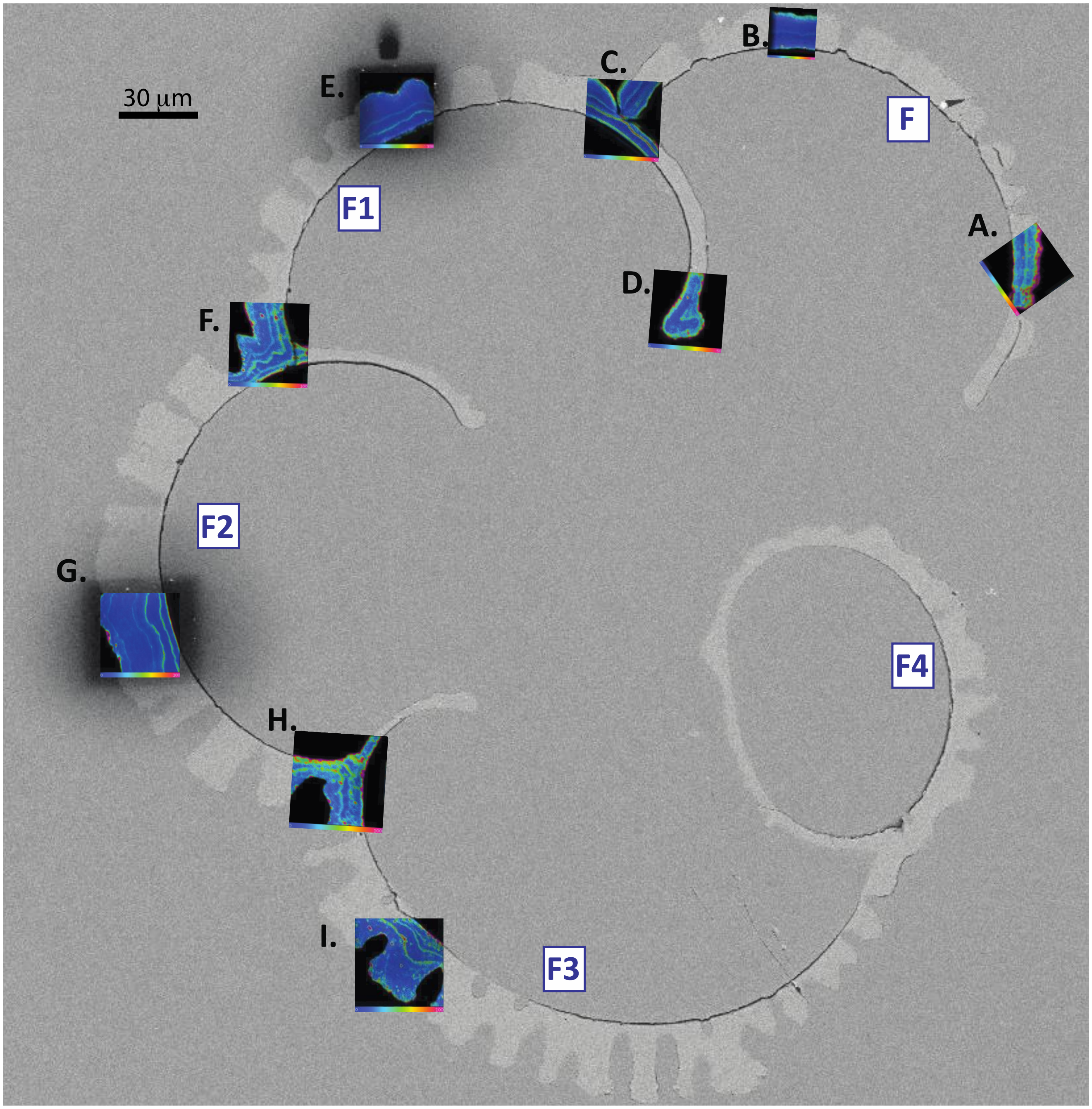

Supplementary Figure 8. SEM of a polished cross-section of specimen 241.
$^{24}Mg/^{40}Ca$ NanoSIMS images are superimposed to identify the locations of the enlarged NanoSIMS images detailed in Supplementary Fig. 9. See Supplementary Fig. 9 for enlarged figures. Note: warm colors = higher ratios. These images represent the summation of a stack of 20-40 NanoSIMS frames that were shift-corrected; the hue scale bar represents the ratio of the summed ^{24}Mg, ^{87}Sr, and/or ^{88}Sr counts divided by ^{40}Ca counts × 10000.

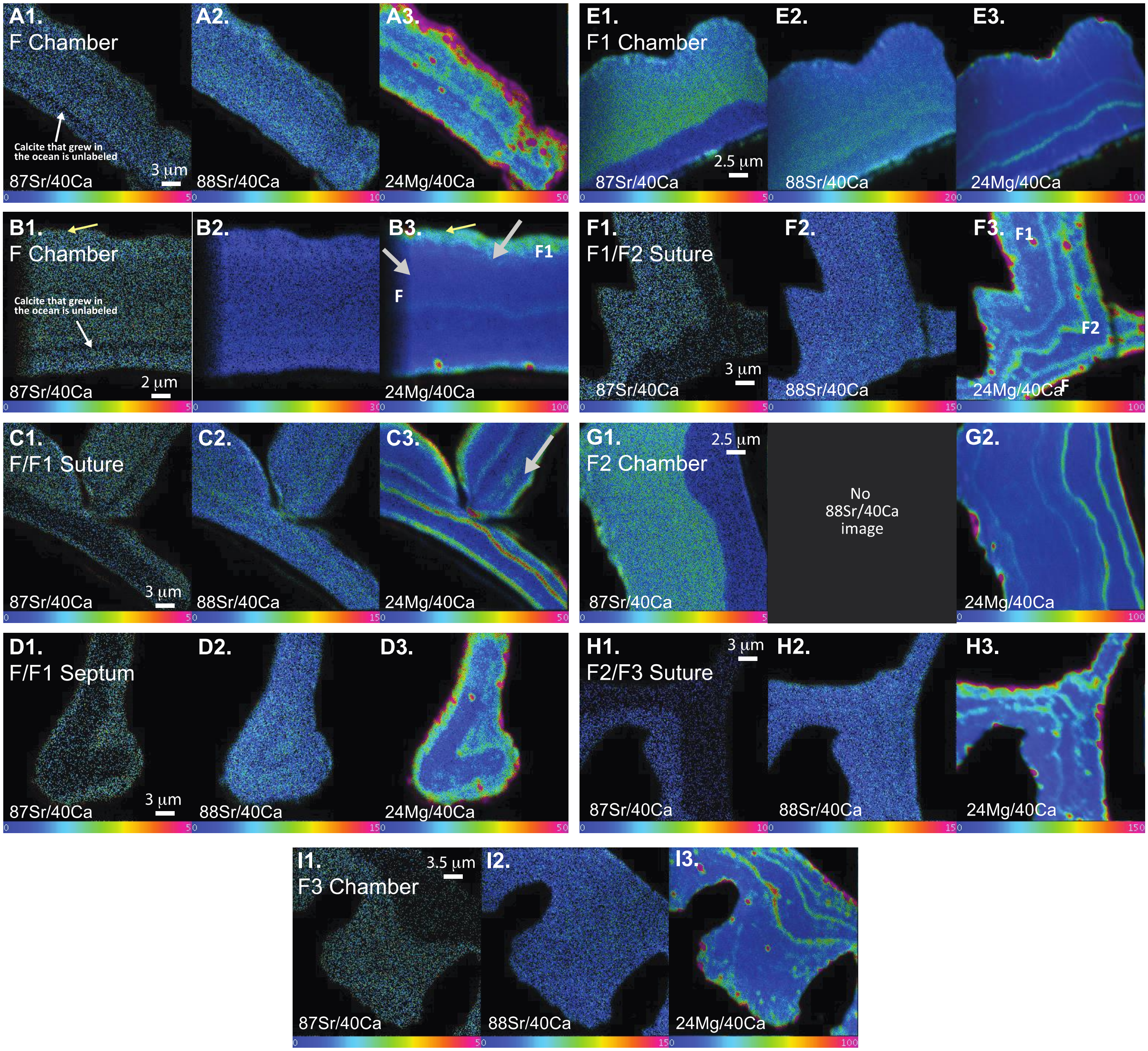

Supplementary Figure 9. Intensity ratio maps of $^{87}Sr/^{40}Ca$, $^{88}Sr/^{40}Ca$, and $^{24}Mg/^{40}Ca$ for Specimen 241. The F chamber was <1 µm thick (Supplementary Fig. 9-A1 through B3) and all other chambers were less than 4-µm thick at the time of collection. As the shell thickened in culture, calcite was added to the outer surface of all previously formed chambers. A thin veneer of calcite precipitated around the septum between the F and F1 chambers (Supplementary Fig. 9-D1 through D3) and on the inner surface of the F1 chamber (less than 1 µm). There is no evidence that calcite was added to the inner surface of the F2 and F3 chambers (e.g. there is no labeled ^{87}Sr calcite). Image letters correspond to the superimposed images on the previous page: (A-B) F chamber. (C) Suture between F and F1 chambers that contains a thin layer of ^{87}Sr-labeled calcite. (D) Septa between F and F1 chambers that contains a thin layer of ^{87}Sr-labeled calcite. (E) F1 chamber. (F) Suture between F1 and F2 chambers. (G) Suture between F1 and F2 chambers. (H) F2 chamber. (I) F3 chamber.

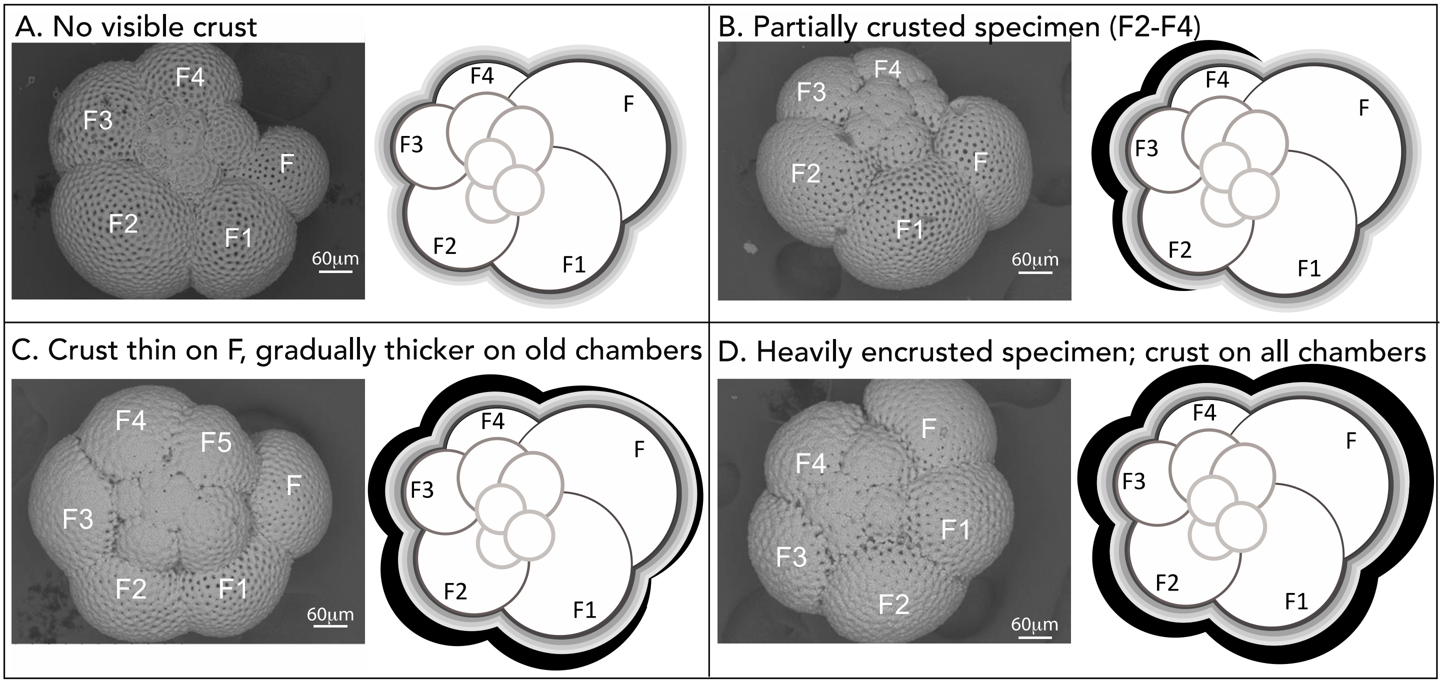

Supplementary Figure 10. SEM images of four fossil *N. dutertrei* displaying various degrees of crusting with a schematic of the ontogenetic and crust growth. Samples were obtained from the core-top interval of core MW91-9 38GGC (2.4 km water depth, 0°N, 159°W, Western Equatorial Pacific). (A) Specimen with no visible crust. The Mg/Ca ratio of all chambers in specimens with no crust is likely similar and may be higher than other specimens among the same sediment sample owing to the absence of the low-Mg/Ca calcite crust. (B) Specimen with no crust on F chamber or F1 chambers; variable crust on older chambers. F3 and F4 are more heavily crusted. (C) Specimen with crust on all chambers, but the crust is thin on the F and F1 chambers and is thicker on older chambers. (D) A heavily crusted specimen with crust on all chambers. The Mg/Ca ratio in all chambers is generally expected to be low and similar owing to the heavy crusted low-Mg ratio calcite crust that is precipitated on the entire shell.

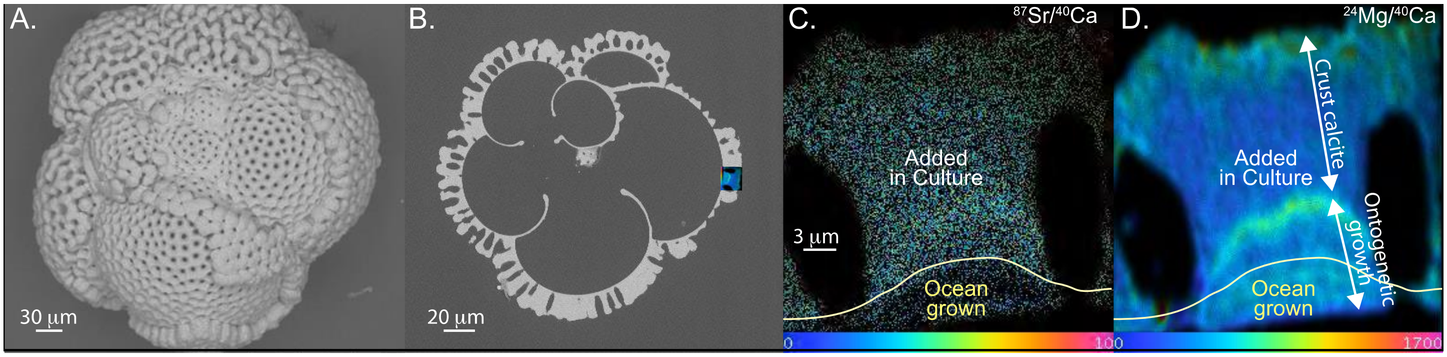

Supplementary Figure 11. SEM and NanoSIMS images of a specimen that began to form crusted calcite in culture. The shell was partially calcified at the time of collection (approximately 3-5 µm thick) and completed its lifecycle in culture. (A) SEM of shell surface with some of the crust broken off revealing the thin ontogenetic calcite. (B) SEM of specimen after embedding in epoxy for NanoSIMS imaging. (C) NanoSIMS image map of the $^{87}Sr/^{40}Ca$. The thin yellow line marks the onset of calcite precipitated in culture, identified by elevated $^{87}Sr/^{40}Ca$ (D) $^{24}Mg/^{40}Ca$ ratios illustrating the outer calcite lacks distinct Mg-banding. These images represent the summation of a stack of 30 nanoSIMS frames that were shift-corrected; the hue scale bar represents the ratio of the summed ^{24}Mg or ^{87}Sr counts divided by ^{40}Ca counts × 10000.
